# Sexually transmitted infections, the silent partner in HIV-infected women in Zimbabwe

**DOI:** 10.4102/sajhivmed.v20i1.849

**Published:** 2019-01-23

**Authors:** Sara Lowe, Tinashe Mudzviti, Ardele Mandiriri, Tinei Shamu, Petronella Mudhokwani, Cleophas Chimbetete, Ruedi Luethy, Margaret Pascoe

**Affiliations:** 1 AIDS Healthcare Foundation, Parirenyatwa Centre of Excellence, Parirenyatwa Hospital, Zimbabwe; 2 Department of Medicine, College of Health Sciences, University of Zimbabwe, Zimbabwe; 3 Newlands Clinic, Zimbabwe; 4 School of Pharmacy, College of Health Sciences, University of Zimbabwe, Zimbabwe

## Abstract

**Background:**

Coinfection rates of HIV and sexually transmitted infections (STIs) are not widely reported in Zimbabwe and no local guidelines regarding the screening of STIs in people living with HIV exist.

**Objectives:**

This cross-sectional study was conducted to determine the prevalence and associated risk factors for STI coinfection in a cohort of HIV-infected women.

**Methods:**

Between January and June 2016, 385 HIV-infected women presenting for routine cervical cancer screening were tested for five STIs: *Neisseria gonorrhoeae* (NG), *Chlamydia trachomatis* (CT), *Trichomonas vaginalis* (TV), Herpes Simplex Virus (HSV) type 2 and *Treponema pallidum* (TP). Socio-demographic characteristics and sexual history were recorded. Multiple logistic regression was used to identify factors associated with the diagnosis of non-viral STIs.

**Results:**

Two hundred and thirty-three participants (60.5%) had a confirmed positive result for at least one STI: HSV 2 prevalence 52.5%, TV 8.1%, CT 2.1%, NG 1.8% and TP 11.4%. Eighty-seven per cent of the women were asymptomatic for any STI; 62.3% of women with a non-viral STI were asymptomatic. Women who had attended tertiary education were 90% less likely to have a non-viral STI (adjusted odds ratio [aOR]: 0.10, 95% confidence interval [CI]: 0.03–0.39, *p* < 0.01). Having more than three lifetime sexual partners was a significant predictor for a non-viral STI diagnosis (aOR: 3.3, 95% CI: 1.5–7.2, *p* < 0.01).

**Conclusion:**

A high prevalence of predominantly asymptomatic STIs is reported in a cohort of HIV-infected women. Syndromic management results in underdiagnosis of asymptomatic patients. More than three lifetime sexual partners and less formal education are risk factors for coinfection with non-viral STI. High-risk women should be screened using aetiological methods.

## Introduction

Sexually transmitted infections (STIs) and their many sequelae are among the top five reasons that adults seek healthcare in low-income settings.^1^ The World Health Organization reports that worldwide more than one million STIs are acquired each day and an estimated 340 million curable bacterial STIs per year. Viral infections constitute a large proportion of prevalent STIs with an estimated 536 million people living with Herpes Simplex Virus (HSV) and 291 million women with Human Papilloma Virus (HPV) infection at any point in time, with the numbers in men likely to be similar.^2^ Adverse events associated with untreated STIs are common: neonatal morbidity and mortality; infertility; ectopic pregnancy and increased risk of transmission of the Human Immunodeficiency Virus (HIV) are all important sequelae. These under recognised STI epidemics constitute a significant global public health threat and have a profound impact on quality of life, particularly among people aged 15–49 years.^3^

HIV and STIs share a complex bidirectional relationship. STIs increase HIV viral shedding in the genital tract, resulting in significant increases in HIV transmission risk. Local inflammation activates HIV replication in the genital compartment independent of HIV in peripheral blood. An individual may have an undetectable plasma HIV viral load (VL), while the genital tract VL is elevated.^4^ It has been shown that the presence of both ulcerative and non-ulcerative STIs significantly increases the risk of both acquiring and transmitting HIV.^5^ In systematic reviews conducted by Fleming and Wasserheit^6^ and Kalichman et al.,^7^ both groups concluded that HIV transmission was facilitated by the presence of other STIs, whether symptomatic or asymptomatic, and that early STI diagnosis and treatment should be part of a high-quality, comprehensive HIV prevention strategy.^6,7,8^

The prevalence of STIs varies according to region, gender and risk group. A number of key populations with high prevalence of STIs have been reported. These include sex workers, their clients and other partners; men who have sex with men; transgender people; people who inject drugs; and people living with HIV (PLWH). Enhanced STI screening is recommended in these key populations.^9,10,11^ However, access and uptake of STI services among these groups is often challenging. In resource-limited settings, there are limited data regarding the prevalence of STIs, with the exception of HIV, which is often the only STI for which functioning surveillance systems are in place. In Zimbabwe, the prevalence of HIV among adults aged 15–64 years is 14.6%, corresponding to approximately 1.2 million PLWH.^12^ Coinfection rates of HIV and STIs are not widely reported and there are no local guidelines regarding the screening for STIs in PLWH. This study was conducted to determine the prevalence of STI coinfection in a cohort of HIV-infected women and to identify associated risk factors for an STI diagnosis.

## Methods

This analytic cross-sectional study was conducted in HIV-infected adult women at Newlands Clinic (NC), Harare, Zimbabwe. NC provides comprehensive HIV care and treatment services to approximately 6000 individuals in the greater Harare urban area. The clinic operates in a public-private partnership with the Ministry of Health and Child Care, Zimbabwe. Funding for the clinic is provided by the Ruedi Luethy Foundation and other partners.^13^ Sexually active, non-pregnant adult women (≥ 18 years of age) attending NC for routine annual cervical screening were invited to participate in the study.

### Study procedures

A questionnaire was verbally administered by a trained study nurse, which collected sociodemographic, medical, gynaecological and sexual history data. Current CD4+ count, HIV VL and antiretroviral therapy (ART) history were documented in the medical history. Current CD4+ count and VL were defined as results which had been obtained within the preceding month. The sexual history included questions regarding age of sexual debut, number of sexual partners, type of sexual activity, STI symptoms, previous STI diagnoses, condom use, family planning, sexual orientation and past history of sexual abuse.

On completion of the questionnaire, a complete abdominal and gynaecological examination including the collection of endocervical swabs was conducted. Findings were recorded on a participant’s respective case report forms. On completion of endocervical swab collection, the nurse proceeded with an examination of the cervix using the visual inspection with acetic acid and cervicography (VIAC) methodology. The swabs were used for *Chlamydia trachomatis* (CT), *Neisseria gonorrhoeae* (NG), and *Trichomonas vaginalis* (TV) using the Cepheid Xpert^®^ CT/NG and Xpert^®^ TV assays.

A 4 mL blood sample was collected into a clot activating tube and processed for onsite testing. Processing involved centrifuging of the sample after clotting and harvesting the serum for subsequent tests. Herpes simplex virus type 2 infection was tested in serum using PreCheck HSV 2 IgG test kits. The seroprevalence of syphilis was defined as having a positive treponemal-specific antibody test using the SD Bioline Rapid Antibody Test with or without a positive non-treponemal RPR carbon assay. All participants with confirmed STI diagnoses were managed using an aetiological approach, and respective antibiotic treatment was administered as recommended in the national guidelines.^14^

VL measurements were performed on EDTA plasma using the Roche COBAS Ampliprep and TaqMan version 2.0, while CD4+ counts were measured in whole blood using a Partec Cyflow Counter II.

### Statistical analysis

Data were entered into a Microsoft Access 2016 database and then exported to Microsoft Excel for cleaning. Cleaned data were exported to Stata 12.1 for analysis. Medians and interquartile ranges (IQR) were used to describe continuous data. A maximum *p*-value of 0.05 was considered statistically significant. Unadjusted odds ratios with 95.0% confidence intervals (CIs) were calculated for risk factors of STIs. Significant risk factors in univariate analysis were further analysed in a multivariable logistic regression to calculate adjusted odds ratios.

## Ethical consideration

The study was approved by the NC Research Unit and the Medical Research Council of Zimbabwe (approval number MRCZ/A/1980). All participants provided written informed consent before enrolling in the study.

## Results

### Participant enrolment

Between 01 January and 30 June 2016, 385 women were enrolled in the study, 356 (93.0%) being on ART. The median age of the participants was 41 years (IQR: 35–47). A total of 171 (44.0%) participants were married and 103 (27.0%) were widows; 86 (22.0%) had seven years or less of education and 57 (15.0%) had reached tertiary education. Table 1 shows the characteristics of the participants in the study.

**TABLE 1:** Participant characteristics (*N* = 385).

Characteristic	Frequency		
	*n*	%	IQR
**Median age, years**	41	-	35–47
**Marital status**			
Married	171	44.4	-
Widowed	103	26.8	-
Divorced/separated	62	16.1	-
Single	49	12.7	-
**CD4+ cell count (cells/μL), median (IQR)**	503	-	347–655
**Viral load**			
< 1000 copies/mL	347	97.5	
< 50 copies/mL	324	91.0	
**Duration on ART, years, median (IQR)**	6.2	-	3.2–9.0
**Sexual history**			
Age at sexual debut, years, median (IQR)	19	-	17–21
Condoms use for last sex	242	62.9	
**HIV status of most recent sexual partner**			
Positive	196	50.0	-
Negative	66	17.1	-
Unknown	123	32.0	-
**Documented previous STI diagnosis**	166	43.1	-
**Reported symptoms of STI**	47	12.2	-
**Reported sexual abuse**	26	6.8	-
**Reported domestic violence**	32	8.3	-

IQR, interquartile range; ART, antiretroviral therapy; STI, sexually transmitted infections.

### HIV treatment history

Of the 356 (93.0%) participants who were taking ART, 324 (91.0%) were virologically suppressed with VLs of < 50 copies/mL. Twelve (3.0%) were severely immunocompromised (CD4+ cell count < 100 cells/μL), but the majority were immunocompetent with the median CD4+ cell count being 503 (IQR: 347–655) cells/μL. Among those receiving ART, the median duration on ART was 6.2 years (IQR: 3.2–9.0) and 323 women (84.0%) were taking a first-line ART regimen.

### Sexually transmitted infection and sexual history

Forty-three percent of the women reported having a previous STI, diagnosed using the syndromic management approach. The majority of patients (*n* = 367, 95.0%) reported having had one or no sex partner in the last six months, while 206 (54.0%) had at least three lifetime partners. All participants reported previous engagement in vaginal sex, while 30 (8.0%) and four (1.0%) also reported oral and anal sex, respectively. Three of the four who engaged in anal sex reported having used condoms. All of the women were heterosexual.

### Sexually transmitted infection prevalence

**FIGURE 1:**
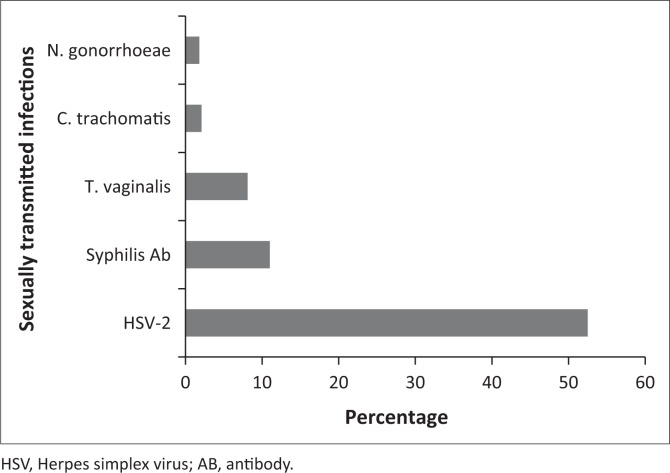
Prevalence of sexually transmitted infections (*n* = 365).

Of the 385 participants screened, 233 (61.0%) women had at least one confirmed result for an STI (HSV-2, TV, NG, CT, syphilis). Seventy-nine (21.0%) women had at least one non-viral STI (TV, NG, CT, syphilis). Eleven (3.0%) women were coinfected with two non-viral STIs: six were positive for syphilis and TV, three for TV and NG and two for TV and CT. Figure 1 shows the percentage of participants diagnosed with each STI. Seropositive HSV 2 prevalence was 52.5%, while TV, CT and NG were 8.1%, 2.1% and 1.8%, respectively. Syphilis was newly diagnosed in 44 (11%) women, of whom 26 (7.0%) were RPR and syphilis antibody test positive, while 18 were positive for syphilis antibody test only. Among the 79 patients with non-viral STIs, 54 (68.0%) did not have signs or symptoms. Among these 54, 31 (57.0%) were positive for syphilis, 6 (11.0%) for CT, 4 (7.0%) for NG and 19 (35.0%) for TV.

### Factors associated with a positive non-viral sexually transmitted infection diagnosis

Women who had attended tertiary education were 90% less likely to have a non-viral STI compared with those without any education (adjusted odds ratio [aOR]: 0.10, 95% CI: 0.03–0.39, *p* < 0.01). Those with ≥ 3 lifetime partners were 3.5 times more likely (aOR: 3.49, 95% CI: 1.64–7.40, *p* < 0.01) to have a non-viral STI compared with those with only one. Women with STI signs and symptoms were three times more likely (aOR: 2.89, 95% CI: 1.54–5.40, *p* < 0.01) to have a non-viral STI than those with no signs and symptoms. Table 2 shows the association of selected risk factors with an STI diagnosis.

**TABLE 2:** Patient characteristics as risk factors for non-viral sexually transmitted infections.

Risk factor	All women *N* = 385		Non-viral STI positive *n* = 79		OR (95% CI, *p*)	aOR (95% CI, *p*)
	*n*	%	*n*	%		
**Age (years)**						
≤ 25	20	5.2	7	35.0	-	-
26–35	80	21.9	22	27.5	0.51 (0.25–1.99, 0.51)	-
> 35	285	78.1	50	17.5	0.39 (0.09–1.04, 0.06)	-
**Education**						
None	24	6.2	9	37.5	-	-
Primary	62	16.1	19	30.7	0.73 (0.27–1.98, 0.54)	0.79 (0.28–2.24, 0.65)
Secondary	242	62.9	47	19.4	**0.40 (0.17–0.97, 0.04)**	0.39 (0.15–1.00, 0.05)
Tertiary	57	14.8	4	7.0	**0.13 (0.03–0.47, < 0.01)**	**0.10 (0.03–0.39, < 0.01)**
**Lifetime partners**						
1	91	23.6	9	9.9	-	-
2	88	22.9	13	14.8	1.57 (0.64–3.90, 0.32)	1.55 (0.60–3.99, 0.37)
≥ 3	206	53.5	57	27.7	**3.49 (1.64–7.40, < 0.01)**	**3.27 (1.49–7.19, < 0.01)**
**Last sex partner type**						
Casual	12	3.1	0	-	-	-
Regular	373	96.9	79	21.2	-	-
**Condom use at last sexual intercourse**						
Yes	246	63.9	53	21.5	-	-
No	139	36.1	26	18.7	0.83 (0.50–1.41, 0.51)	-
**STI signs and symptoms**						
No	312	81.0	54	17.3	-	
Yes	73	19.0	25	34.3	**2.49 (1.41–4.38, < 0.01)**	**2.89 (1.54–5.40, < 0.01)**
**Previous STI**						
No	219	56.9	39	17.8	-	-
Yes	166	43.1	40	24.1	1.46 (0.89–2.40, 0.13)	-
**Age at sexual debut (years)**						
< 16	25	6.5	7	28.0	-	-
16–20	237	61.6	59	24.9	0.85 (0.34–2.14, 0.73)	1.30 (0.49–3.51, 0.60)
**> 20**	123	31.9	13	10.6	**0.39 (0.16–0.93, 0.03)**	0.60 (0.19–1.86, 0.37)
**Sexual abuse or domestic violence**						
No	333	86.5	68	(86.1	-	-
Yes	52	13.5	11	13.9	1.05 (0.51–2.14, 0.90)	-

OR, odds ratio; CI, confidence interval; STI, sexually transmitted infection.

Note: Bold values indicate statistically significant, *p*-value < 0.05

## Discussion

Our study reports a high prevalence of STIs in a cohort of HIV-infected women in Zimbabwe. Sixty-one per cent of women had a positive confirmatory test for any STI and approximately one in five women was diagnosed with a treatable non-viral STI. The majority of the women in this study (62.0%) were asymptomatic at the time of diagnosis of non-viral STIs and would therefore not have received treatment using the current syndromic management guidelines. A wide variation in STI prevalence data collected mostly through antenatal programmes has been presented across different low- and middle-income countries.^15^ In a similar study conducted in Khartoum, Sudan, only 17.7% of the attending antenatal clinics were found to harbour at least one non-viral STI (TV, NG, CT, TP). All the participants in the Khartoum study were HIV-negative.^16^ Untreated symptomatic or asymptomatic STIs are not only associated with significant morbidity, but are also associated with an increased risk of transmission of HIV.^8^ The presence of both ulcerative and non-ulcerative STIs has been associated with increased concentrations of HIV RNA in mucosal secretions, plasma and decreasing CD4+ cell counts.^7^ Identifying these hidden infections with thorough sexual history taking, genital examination and aetiological diagnosis has both individual and public health benefits.

Reported syphilis prevalence in this study is significantly higher (11.0%) than what has been previously described in Zimbabwean cohorts. Gwanzura et al. reported a prevalence of 2.3% in male factory workers. The prevalence of active syphilis in HIV-infected women and all antenatal clinic attendees has been previously reported at 4.0% and 1.2%, respectively.^17,18,19^ This increase is in keeping with global trends of syphilis incidence and higher prevalence in PLWH.^7,20^ Rekart et al. suggest a novel hypothesis that ART may potentially alter both innate and acquired immune responses in ways that may lead to enhanced susceptibility to syphilis.^21^ With a larger proportion of patients now taking ART (93.0% in this study), this enhanced susceptibility could be a significant factor contributing to the rise in incident syphilis in HIV-infected treated adults. The high prevalence of syphilis in our study provides further evidence to support the need to enhance the surveillance of syphilis and provide effective syphilis control programmes within existing HIV care and treatment programmes.

The second most prevalent non-viral STI in this study was TV, which was confirmed in 8.0% of the participants. Similar to other STIs, it is increasingly recognised that TV plays an important role in increasing the risk of both acquisition and onward transmission of HIV. Using mathematical models, Quinlivan et al. estimated that 22.0% of projected HIV transmissions from women in the United States are attributable to TV infections and up to 2.0% of all HIV transmissions in the United States may be related to TV infection.^22^

The prevalence of infection with chlamydia varies significantly between regions, countries and risk groups. The prevalence of NG and CT in our study was 1.8% and 2.1%, respectively. Studies conducted in Kenya, Zimbabwe, Nigeria and South Africa have shown that the prevalence of CT varied between 6.0% and 20.0% in these countries.^23,24,25^ A Zimbabwean study investigating the prevalence of NG and CT in 5448 men and women congregating at bottle stores revealed a baseline prevalence of 2.2% and 3.8%, respectively. The HIV status of these individuals was, however, not recorded. The factors associated with incident infection were being female and having more than one sexual partner. The proportion of patients with incident infection not reporting symptoms was greater than 75.0% for both infections;^26,27^ this is consistent with other studies in PLWH in the region.

An HSV 2 seropositivity rate of 52.0% is in keeping with global published rates in PLWH of 50.0% – 90.0%.^27^ Data from a review conducted by Smith and Robinson showed that up to 90.0% of PLWH in some settings were coinfected with HSV-2.^27^ Herpes Simplex Virus type 2and HIV are synergistic co-pathogens. The parallel intersecting epidemics of the HIV and HSV infections are well documented, and studies indicate that HSV 2 plays an important role in the spread of HIV and affects virological control of the coinfected untreated patient.^28^

Analysis from this study suggests that women with less formal education were more likely to be diagnosed with an STI; this is consistent with findings from other similar studies. Quinlivan et al. found a five-fold increase in risk of infection with TV in women living with HIV with low reported education status.^22^ Suggested explanations for this commonly reported observation are that lesser educated women are less likely to be employed and therefore are more dependent on their sexual partners or more likely to engage in transactional sex. Negotiating safe sex in both these situations may be more challenging.^29,30^

Multivariate analysis revealed that women reporting more than three lifetime sexual partners were three times as likely to be diagnosed with a non-viral STI. Taking a thorough sexual history is an important tool in risk profiling of patients, and in countries where resources are limited, it can enable healthcare professionals to target high-risk patients for targeted screening. Participants in this study were not recruited based on symptoms. However, on completion of a detailed sexual history, 13.0% of women reported ongoing symptoms consistent with an STI diagnosis. Possible reasons for participants delaying in seeking treatment include stigma associated with an STI diagnosis.^31^ The level of education attained by an individual may also have a bearing on whether they will seek treatment as they may not be in a position to relate ongoing symptoms to a possible STI diagnosis. This is of importance in countries like Zimbabwe where STI treatment programmes focus on syndromic management approach.

The prevalence of previous or current sexual abuse and/or intimate partner violence (IPV) was reported in this study. Our results are consistent with published data reporting a strong association between IPV and HIV infection.^32^ Studies from Tanzania and Rwanda report that women experiencing IPV are up to three times more likely to acquire HIV.^33,34^ Studies also report an association between sexual abuse in childhood and increased sexual risk behaviours in adulthood leading to higher rates of STIs, including HIV.^35^ Importantly, many of the women in the study had not previously disclosed this sensitive information despite long-term and recurrent attendance at a health facility, highlighting the need for confidential routine sexual history taking in all HIV-infected women.

This study highlights an unrecognised burden of STIs and defines an at-risk population of HIV-infected women. An important limitation of the study is, however, the lack of generalisability of our results to all HIV-infected women, as participants were recruited from one urban site. The cohort also did not include key populations such as sex workers or adolescents and young adults. The investigators did not survey any additional anatomical sites; consequently, the results for this study focus on vaginal and cervical STIs (except for syphilis). Women were asked to report historical events which may introduce recall bias particularly relating to number of sexual partners and condom use.

In conclusion, we have reported a high prevalence of non-viral STIs in a cohort of largely asymptomatic HIV-infected women. Those reporting more than three lifetime partners and with less formal education were at higher risk of STI acquisition. The major strategy in most resource-poor settings for STI control is focused on syndromic management of genitourinary infections. Although we recognise this is an important public health measure, this hidden epidemic may be associated with significant morbidity and drives onward HIV transmission. STI control remains an important aspect of HIV prevention. These results emphasise the need for identification of high-risk women through routine sexual history checking and targeted aetiological screening of high-risk individuals for asymptomatic STIs. There is an increased role for diagnostic technology which can be used for surveillance and STI screening of at-risk women. Polymerase chain reaction and point-of-care diagnostic technology need to be included as part of rapid STI treatment modalities.
